# Pharmacokinetic/Pharmacodynamic Model of Neutropenia in Real-Life Palbociclib-Treated Patients

**DOI:** 10.3390/pharmaceutics13101708

**Published:** 2021-10-16

**Authors:** Alexandre Le Marouille, Emma Petit, Courèche Kaderbhaï, Isabelle Desmoulins, Audrey Hennequin, Didier Mayeur, Jean-David Fumet, Sylvain Ladoire, Zoé Tharin, Siavoshe Ayati, Silvia Ilie, Bernard Royer, Antonin Schmitt

**Affiliations:** 1 INSERM U1231, School of Medicine and Pharmacy, University of Burgundy Franche-Comté, 21000 Dijon, France; alemarouille@cgfl.fr (A.L.M.); emma.petit@edu.univ-fcomte.fr (E.P.); jdfumet@cgfl.fr (J.-D.F.); sladoire@cgfl.fr (S.L.); 2 Centre Georges-François Leclerc, Oncology Department, 21000 Dijon, France; cgkaderbhai@cgfl.fr (C.K.); idesmoulins@cgfl.fr (I.D.); ahennequin@cgfl.fr (A.H.); dmayeur@cgfl.fr (D.M.); ztharin@cgfl.fr (Z.T.); sayati@cgfl.fr (S.A.); silie@cgfl.fr (S.I.); 3 Laboratoire de Pharmacologie Clinique et Toxicologie, CHU Besançon, 25000 Besançon, France; broyer@chu-besancon.fr; 4 INSERM, EFS BFC, UMR1098, RIGHT, Interactions Greffon-Hôte-Tumeur/Ingénierie Cellulaire et Génique, School of Medicine and Pharmacy, University of Bourgogne Franche-Comté, 25000 Besançon, France; 5 Centre Georges-François Leclerc, Pharmacy Department, 21000 Dijon, France

**Keywords:** palbociclib, neutropenia, pharmacokinetic/pharmacodynamic

## Abstract

Palbociclib is an oral CDK4/6 inhibitor indicated in HR+/HER2- advanced or metastatic breast cancer in combination with hormonotherapy. Its main toxicity is neutropenia. The aim of our study was to describe the kinetics of circulating neutrophils from real-life palbociclib-treated patients. A population pharmacokinetic (popPK) model was first constructed to describe palbociclib pharmacokinetic (PK). Individual PK parameters obtained were then used in the pharmacokinetic/pharmacodynamic (PK/PD) model to depict the relation between palbociclib concentrations and absolute neutrophil counts (ANC). The models were built with a population of 143 patients. Palbociclib samples were routinely collected during therapeutic drug monitoring, whereas ANC were retrospectively retrieved from the patient files. The optimal popPK model was a mono-compartmental model with a first-order absorption constant of 0.187 h^−1^ and an apparent clearance Cl/F of 57.09 L (32.8% of inter individuality variability (IIV)). The apparent volume of distribution (1580 L) and the lag-time (T_lag_: 0.658 h) were fixed to values from the literature. An increase in creatinine clearance and a decrease in alkaline phosphatase led to an increase in palbociclib Cl/F. To describe ANC kinetics during treatment, Friberg’s PK/PD model, with linear drug effect, was used. Parameters estimated were Base (2.92 G/L; 29.6% IIV), Slope (0.0011 L/µg; 28.8% IIV), Mean Transit Time (MTT; 5.29 days; 17.9% IIV) and γ (0.102). The only significant covariate was age on the initial ANC (Base), with lower ANC in younger patients. PK/PD model-based simulations show that the higher the estimated C_ressSS_ (trough concentration at steady state), the higher the risk of developing neutropenia. In order to present a risk lower than 20% to developing a grade 4 neutropenia, the patient should show an estimated C_ressSS_ lower than 100 µg/L.

## 1. Introduction

Palbociclib is an oral inhibitor of cyclin 4 and 6 dependent kinases (CDK4/6) used in HR+/HER2- breast cancer [1,2,3,4,5]. An absolute gain of 10.3 and 5.4 months in progression-free survival was observed, respectively, in patients treated with palbociclib and letrozole compared to letrozole alone, and in patients treated with palbociclib and fulvestrant compared to fulvestrant alone in patients previously treated with hormone therapy. The recommended dose of palbociclib is 125 mg per os once daily for 21 days out of 28. Treatment continues until progression or serious adverse events. Dosage adjustments are made based on the occurrence of adverse events [3,5]. The most common adverse event is neutropenia [1,2,3,4,5,6,7]. To avoid complications, at the beginning of treatment with palbociclib and at the beginning of each cycle, as well as on day 15 of the first two treatment cycles, a blood count is performed. An absolute neutrophil count (ANC) ≥ 1000/mm^3^ (N: 1700 to 7500/mm^3^) is recommended to start/continue the treatment [3,5].

Two pharmacokinetic/pharmacodynamic (PK/PD) models describe the relationship between palbociclib and ANC kinetics. The first model developed by Sun et al. [8] is a population PK/PD model based on palbociclib phase II and III data. This model, derived from PK/PD models used for cytotoxic drugs (i.e., Friberg’s model [9]), is the first PK/PD model applied to palbociclib in humans. It showed the impact of age, weight, and food effect on the pharmacokinetics (PK) of palbociclib and the impact of gender and albuminemia level on ANC kinetics. The second model, developed by Chen et al. [10], is a preclinical PK/PD model, constructed from in vitro data and using PK/PD data from endogenous G-CSF (granulocyte colony stimulating factor) studies. The authors used several concentrations of palbociclib on cell cultures to describe the effect of palbociclib on granulocyte stem cells. An inhibitory effect (i.e., by an inhibition effect of stem cells entering the S phase and a stimulation effect of stem cells entering the G0 phase) is modeled in order to mimic the palbociclib effect on the proliferation of the bone marrow stem cells.

The aim of the present work was to describe the PK of palbociclib and to model the PK/PD relationship between long-term kinetics of ANC and palbociclib concentrations, in the general population, based on data from real-life follow-up. Additionally, based on those models, simulations were conducted to determine the optimal exposure that limits the risk of neutropenia to a reasonable one.

## 2. Materials and Methods

### 2.1. Patients and Sampling

All consecutive patients included in the study from 28 October 2018 to 15 March 2021 received palbociclib as breast cancer (RH+/HER2- or HER2+ non-amplified) treatment in our institution. Patients with incomplete dosing history or insufficient ANC at the period of the study were not included in the PKPD part of the study (minimum of 3 ANC’s samples per patient). Patient data were retrieved retrospectively from the patient record management software. Palbociclib therapeutic drug monitoring is routinely performed in our institution. Thus, blood samples to quantify patient palbociclib exposure were available. Additionally, ANC were extracted from biology reports completed during treatment as required by the marketing authorization or during any patients’ hospitalizations, if deemed appropriate, during the first treatment year.

No specific informed consent was required, as samples were regular. However, a general consent was signed by patients stipulating that its data may anonymously be used for research purposes. Thus, data used in this manuscript were recorded in such a manner that confidentiality was ensured following these guidelines. Additionally, our protocol of analyses was approved by our Institutional Review Board and was in accordance with the Declaration of Helsinki.

### 2.2. Analytical Methods of Palbociclib

Palbociclib blood samples were analyzed by liquid chromatography-mass spectrometry using the method described by Jolibois et al. [11] which allows the simultaneous measurement of palbociclib, olaparib, cabozantinib, pazopanib, sorafenib, sunitinib and desethyl-sunitinib. After blood collection, samples were rapidly centrifuged and frozen until the analysis. Sample extraction was performed as follows: 150 µL of human plasma and 100 µL of NaOH were added to 10 µL of an internal standard solution (containing isotopic palbociclib, olaparib, cabozantinib, pazopanib, sorafenib and sunitinib). Samples were vortexed for a few seconds, then 700 µL of ethyl acetate was added. After being vortexed for 30 s, the samples were centrifuged at 10,000 RPM for 5 min. In an extraction tube, 600 µL of supernatant were collected and evaporated under nitrogen flow. The dried samples were reconstituted with 150 µL of a mixture of 55% of methanol/45% of solvent A (ammonium acetate 1 M in water, pH adjusted to 3.2 with formic acid). The samples were vortexed for 30 s and filtrated using Captiva ND Lipids plates and assayed with an LC-MS/MS device. With this method, the lower limit of quantification (LLOQ) of palbociclib was 6 ng/mL, and the between-run and within-run accuracy and precision were lower than 13.3% and 12.9% for the lowest level control, 7.5% and 10.0% for the medium level control and 2.8% and 5.0% for the highest level control, respectively.

### 2.3. Population Pharmacokinetic Model

Several population pharmacokinetic (popPK) models have been tested. The base model was a 1-compartmental model with first-order absorption and elimination clearance. Other models were tested such as with an absorption lag-time or a 2-compartmental model. Residual error was described either with an additive, a proportional or a combined error model.

### 2.4. Pharmacokinetic/Pharmacodynamic Model

The aim of this PK/PD model is to describe the relationship between the plasma concentrations of palbociclib and the ANC kinetic during the first year of treatment. Individual pharmacokinetic parameters were estimated with the popPK model and used as individual constants (regressors in Monolix^®^) in the model in order to compute palbociclib concentrations.

**Figure 1:**
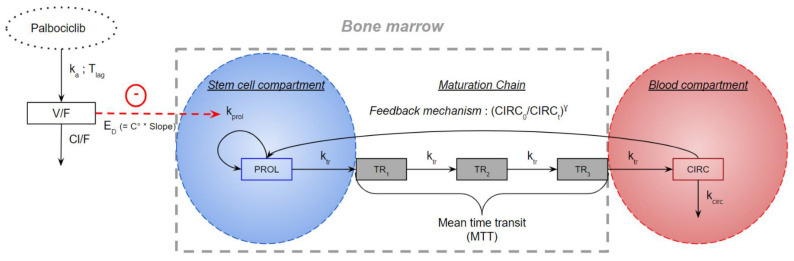
Pharmacokinetic/pharmacodynamic model. C°: concentration of palbociclib; CIRC: compartment corresponding to the circulating neutrophils; CIRC_0_: ANC at time 0 in CIRC compartment; CIRC_t_: ANC at time t in CIRC compartment; Cl/F: palbociclib apparent clearance; E_D_: drug effect; γ: feedback mechanism; k_a_: palbociclib absorption constant; k_circ_: rate of elimination of neutrophils from the systemic circulation; k_prol_: rate of stem cell proliferation; k_tr_: maturation rate; MTT: mean transit time; PROL: proliferation compartment; Slope: sensitivity to palbociclib-induced neutropenia; T_lag_: palbociclib absorption lag time; TR_X_: transit compartments; V/F: palbociclib apparent volume of distribution.

The structural model used as base is the one initially developed by Friberg et al. [9] to describe cytotoxic-induced neutropenia. This model is a semi-mechanistic model mimicking neutropoiesis (Figure 1).

This model is composed of five compartments. First, a PROL compartment, which is assumed to contain stem cells that will become neutrophils after maturation via three transit compartments (TR_1_, TR_2_ and TR_3_). Lastly, a CIRC compartment corresponding to the circulating neutrophils.

The endogenous effect of G-CSF is represented by a feedback mechanism γ. The drug effect (E_D_) is a linear proportionality constant relating palbociclib concentration to its effect on stem cells in the PROL compartment. It represents individual sensitivity to palbociclib-induced neutropenia. The mean transit time (MTT) is the average time it takes for a stem cell to reach the CIRC compartment. k_tr_ is equal to the number of compartments plus one (i.e., 3 + 1) divided by MTT. The rate of stem cell proliferation is k_prol_. The rate of elimination of neutrophils from the systemic circulation is k_circ_. In most applications of Friberg’s model, it is assumed that the transfer constant (k_tr_) is equal to the proliferation constant (k_prol_) which is equal to the elimination constant of circulating neutrophils (k_circ_). This simplifies the calculations and reduces the analysis time. At baseline, compartments PROL, TR_1_, TR_2_, TR_3_ and CIRC are equal to the same value: Base. The ANC were log-transformed to facilitate the estimation of parameters. An additive residual error was used on log-transformed values (i.e., corresponding to a proportional error model on non-log-transformed data).

Patients without ANC samples or with incomplete follow-up history were not included in the analyses.

### 2.5. Estimation of Model Parameters and Software Used

To develop the popPK and the PK/PD model, a nonlinear mixed effects regression approach was used. For this step, the Monolix^®^ software version 2020R1 was used (Lixoft SAS, Antony, France). The estimation of the population parameters was performed using the SAEM (Stochastic Approximation Expectation-Maximization) algorithm. The interindividual variability (IIV) was coded as $Param_{i}=Param_{pop}\times e^{\eta }{}^{\;}$ with *Param_i_* the individual parameter value, *Param_pop_* the typical population value, and *η* the random effect that follows a normal distribution centered on 0 and of standard deviation *ω*. The result of this random effect is given as the coefficient of variation (CV in %) which is equal to: $\;CV=\sqrt{e^{\omega ²}-1}$ .

### 2.6. Covariate

For population PK analysis, covariates were selected based on routinely available data and physiological considerations. Continuous covariates selected for the popPK model were age, weight, creatininemia, creatinine clearance (Clcr) according to the Cockcroft–Gault formula, aspartate aminotransferase (ASAT), alanine aminotransferase (ALAT), γ-glutamyltranspeptidase (γ-GT), lactate dehydrogenase, total bilirubinemia, alkaline phosphatase (ALP) and albuminemia, whereas concurrent hormonotherapy (fulvestrant/letrozole/others/NK (not known)) was the only categorical covariate.

For PK/PD analysis, covariates were selected based on previous knowledge [8,9,12]. Continuous selected covariates for the PK/PD model were age and albuminemia. Selected categorical covariates were long-term hormonotherapy (yes/no/NK), previous radiotherapy (yes/no/NK), presence of metastases (yes/no/NK), concurrent hormonotherapy (fulvestrant/letrozole/others/NK), and the number of previous lines of chemotherapy.

Missing covariates were replaced by the median value in our population.

The selection of covariates was undertaken in the same way for the popPK and the PK/PD model. Covariates were plotted against random effect from the base models (i.e., models without covariates) in order to search for any tendency. Additionally, Wald tests were performed. If the Wald test was significant (*p* < 0.05) or if the graphical approach shows a relationship between the random effect and a covariate, a forward/backward approach was conducted [13]. Significant covariates were then included independently in the model. A covariate was considered relevant if a decrease in the objective function value (OFV) of 3.84 points (significant at 0.05) was observed. All covariates leading to an improvement in the OFV were then added to the model, leading to a full covariate model. Covariates were then excluded one by one and change in OFV was monitored. A covariate was considered relevant if an increase in the OFV of 6.64 points (significant at 0.01) was observed after its removal. The final model is the one with all covariates considered relevant after forward/backward steps.

The effect of continuous covariates was defined as follows:

(1) $$Param_{i}=Param_{pop}\times {\left(\frac{COV_{i}}{COV_{med}}\right)}^{\beta_{COV}}$$

With Param_i_ the individual parameter, *Param_pop_* the typical value of the population parameter, *COV_med_* the median value of the covariate in the population, *COV_i_* the value of the individual covariate, *β_cov_* the impact of the covariate.

For categorical covariates, one class will be considered the reference class:

(2) $$Param_{i}=Param_{pop}\times e^{\beta *covi^{\;}}$$

With *Param_i_* the individual parameter, *Param_pop_* the typical value of the population parameter, *Cov_i_* the covariate coded as 0 (reference class) and 1 and β the impact of the covariate.

### 2.7. Model Evaluation

Final model selection was based on comparison of the models’ OFV, the relative standard error (RSE%, i.e., precision) of the parameter estimates, the ability of Monolix^®^ to converge a parameter to a typical population value (i.e., if the model estimates the same parameter value after several runs), and graphical diagnostics plots, including observations versus individual predictions plots, and residual errors plots (individual residuals versus time or observed concentrations). The control of normalized prediction distribution errors (NDPE), numerical predictive checks and corrected visual checks were also performed.

Observations versus individual predictions plot visualizes the fit of the model to the data used. The RSE allow for judgement of the stability of the final model.

### 2.8. Simulation

Using Simulx2020R1^®^ software, the impact of covariates on ANC kinetics was explored using simulation of the final PK/PD model. The simulations were run over 100 days with 28-day cycles (21 days with 125 mg palbociclib daily followed by 7 days off). Each covariate was set to either its median value or its minimum and maximum. IIV were not considered.

In addition, 5,000 ANC time courses were computed according to each C_resSS_. The proportion of patients with at least grade 3 and the proportion of patients with grade 4 neutropenia was then calculated.

### 2.9. Palbociclib Exposure vs. Dose Reduction and Cut-Off Estimation

Trough palbociclib concentrations at steady state (C_resSS_) during the first cycle were estimated via the final popPK model for each patient. Palbociclib areas under the curve of concentration time course (AUC in mg.h/L) for a single dose were computed according to the relation AUC = Dose/Cl (with the Dose, the dose at initiation in mg, Cl, the individual clearance estimated via the final popPK model in L/h). Percentage of patients with dose reduction was computed for 4 groups of patients according to their exposure metrics values (AUC or C_resSS_). Only patients who started treatment at full dose (i.e., 125 mg/d) and with complete intake and follow-up history were included.

## 3. Results

### 3.1. Population Description

All of our patients were female and their characteristics are presented in Table 1 and Table 2. In the construction of the popPK model, 181 samples from 143 unique patients were used. One hundred and eight patients had one PK sample taken, 32 patients had two different PK samples, and 3 patients had three different PK samples. Samples from the same patient were considered as independent.

Palbociclib samples were collected between day 1 and 28 after the start of the cycle. Of these, 31 samples (18%) were collected during the first 8 days. The samples were collected between 0.9 and 197.25 h after the last administration of palbociclib. The mean concentration was 77 (±80.2) µg/L, while concentrations ranged from 6 (LLOQ) to 229 µg/L. No concentration was below the LLOQ. Doses administered during the PK evaluation were 75 mg (*n* = 16), 100 mg (*n* = 38), and 125 mg (*n* = 127) per day, 21 days out of 28.

### 3.2. Population Pharmacokinetic Model

The model that best described the PK data was a mono-compartmental model with an absorption lag-time (T_lag_), an absorption rate (k_a_), and an additive error model. The lack of early observations (4% of the samples were taken 2 h maximum after the last intake) did not allow us to estimate T_lag_ and V/F, so they were fixed to the values of a mono-compartmental model of palbociclib developed by Royer et al. [14]. IIV was added only on Cl/F, as when other IIV were added, the estimations were not sufficiently precise. Parameter values estimated by Monolix^®^ are presented in Table 3. The RSE are below 40%, showing a good stability of the model.

Construction of a bicompartmental model was not possible, as the model failed to converge on typical second-compartment population parameters (second-compartment volume and inter-compartment clearance).

Covariates tested according to graphical approach and/or Wald test were creatininemia, creatinine clearance (according to the Cockcroft–Gault formula), age, weight, and ALP concentration. Fourteen patients had missing covariates, so they had the median value for our population. Only two covariates significantly decreased OFV after forward/backward approach: Cl_cr_ and ALP concentration on apparent clearance (Cl/F). Their addition resulted in a decrease in OFV of 41.38 points and a decrease in Cl/F IIV of 8.8%.

The numerical predictive checks (Supplementary Figure S1) showed a good adequacy of the model with our data. The cumulative distribution function and the probability density function of the NDPE also showed a reasonable agreement (Supplementary Figures S2 and S3).

**Figure 2:**
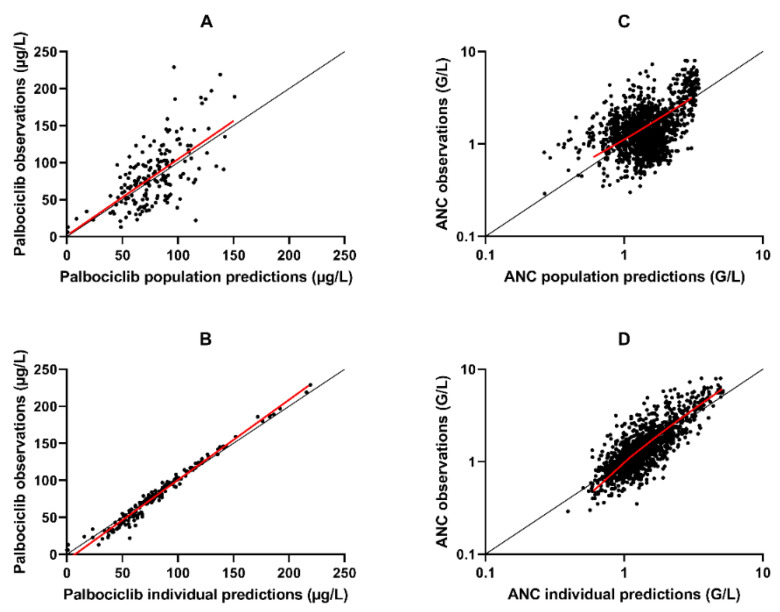
Observations versus population or individual predictions. (**A**) Palbociclib observations versus palbociclib population predictions of the popPK model. (**B**) Palbociclib observations versus palbociclib individual predictions of the popPK model. (**C**) ANC observations versus ANC population predictions of the PK/PD model (log scale). (**D**) ANC observations versus ANC individual predictions of the PK/PD model (log scale). The x-axis represents the population or individual predictions, and the y-axis represents the observations. The red line (solid line) represents the trend of the points. The black line represents the y = x line.

The Figure 2A,B shows a good fit of the final popPK model to the data. The population predictions versus observed concentration did not show any particular mismatch, whereas a slight under-prediction of high values and an over-prediction of low values is observed when individual predictions are compared to observed palbociclib concentrations.

### 3.3. Pharmacokinetic/Pharmacodynamic Model

Of the 143 patients used for the popPK model, 15 patients could not be used for the PK/PD analysis: 10 patients without ANC samples in our database and 5 patients with incomplete intake and follow-up history (but not related to toxicity).

PK/PD patients’ characteristics are presented in Table 1 and Table 2. One thousand five hundred and eight ANC were recovered, i.e., an average of 11.8 ANC per patient with a minimum of 3 and a maximum of 41. The duration of follow-up ranged from 35 days to 1 year (13 cycles). ANC values ranged from 0.29 G/L to 8 G/L. One point six percent of samples had a value below 0.5 G/L, 25.7% between 0.5 and 1 G/L, 50.8% between 1 and 2 G/L and 21.9% above 2 G/L.

Several semi-mechanistic models were tested, such as Friberg’s model with an E_max_ effect, a model with more complex G-CSF effect and a model with quiescent compartments of stem cells. The most relevant model was the one developed by Friberg with a linear drug effect. During the model building phases, Monolix^®^ failed to converge to a value for gamma variability. By removing this variability, the OFV did not change significantly (<3.84), so this variability was removed. The parameters estimated by Monolix^®^ are presented in Table 4. The RSE of the population parameters are less than 40% showing good model stability.

Covariates significant in the forward step were age and albuminemia on Base, but only age was significant in the backward step. Age decreased the OFV of the final model by 9.47 points and the baseline IIV by 2.2%. The median parameters of the 100 bootstrap simulations are close to the parameters estimated in the final PK/PD model (Table 4).

The observations versus individual predictions plot (Figure 2C,D) comparing observations to predictions shows a slight underestimation of the highest values (> 2.5 G/L) and an overestimation of lowest values (< 0.6 G/L).

The corrected visual predictive checks (Supplementary Figure S4) showed that the model gave a good description of the ANC during the first year of treatment with palbociclib. The cumulative distribution function and the probability density function of the NDPE showed a good distribution of the empirical NDPE of the PK/PD model (Supplementary Figures S5 and S6).

### 3.4. Simulation

**Figure 3:**
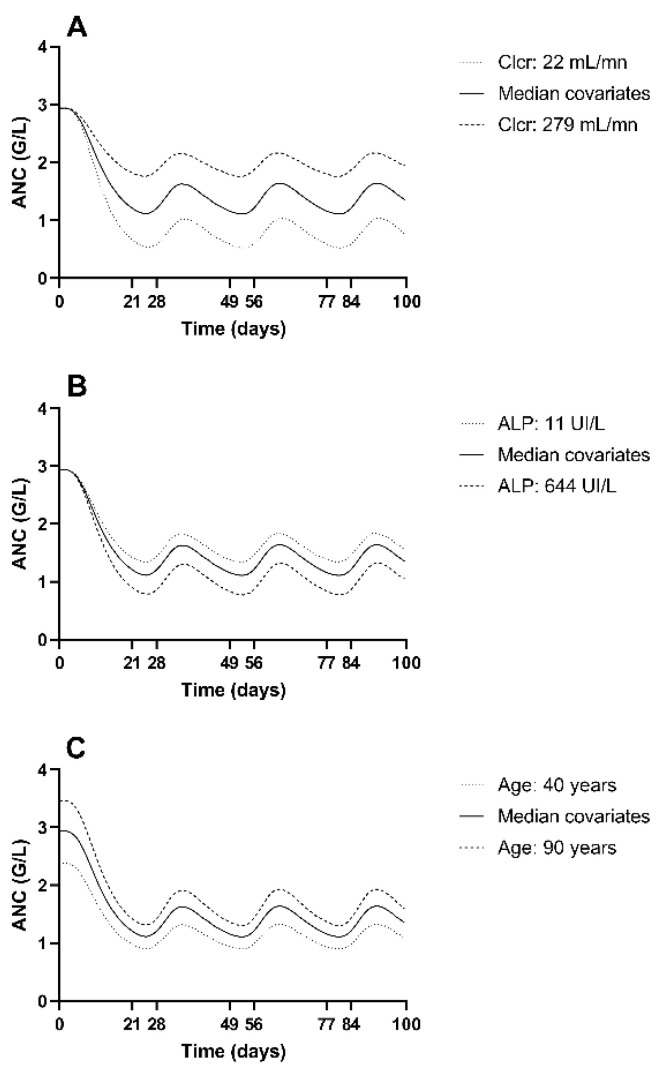
Simulation of the impact of different covariates’ values on ANC time course. (**A**) Simulations of palbociclib treatment with median covariates except Cl_cr_. (**B**) Simulations of palbociclib treatment with median covariates except ALP. (**C**) Simulations of palbociclib treatment with median covariates except age. Cycle of 21 days of treatment (125 mg of palbociclib per day), then 7 days of therapeutic pause. In each graph, only one covariate is changed, the others are fixed at the median value. The extreme values of the covariates in the population were selected for each simulation.

Looking at the different simulations of ANC kinetics as a function of covariates (Figure 3), we observe that a “median” patient (i.e., with median covariates) does not experience neutropenia. Decreased Cl_cr_ or increased ALP leads to an increase in plasma concentrations and therefore in the neutropenic effect. According to our model, the younger a patient, the lower her baseline ANC. Consequently, younger patients are more at risk of neutropenia.

**Figure 4:**
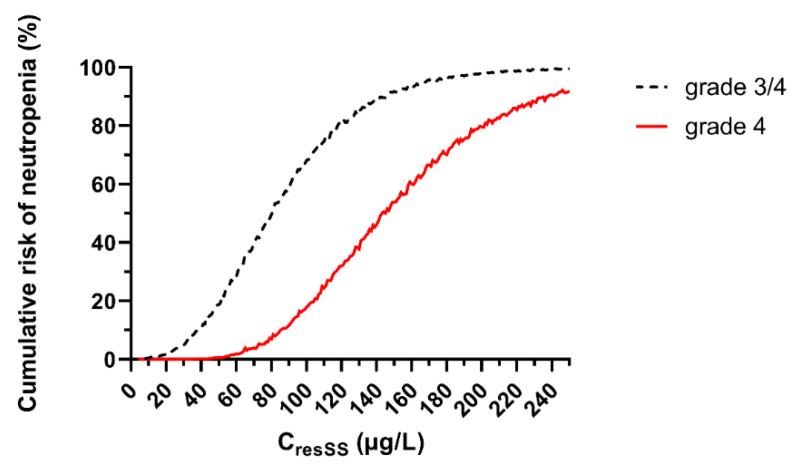
Risk of grade 3 or grade 3/4 neutropenia depending on the residual concentration at steady state. 5000 simulations of ANC kinetics were simulated at every C_resSS_ to 0 at 250 µg/L.

The risk of developing a grade 3 or 4 neutropenia at the nadir increases with estimated C_resSS_ (Figure 4). At an estimated C_resSS_ of 100 µg/L, a patient has an 18% risk of developing grade 4 neutropenia (Figure 4).

### 3.5. Palbociclib Exposure vs. Dose Reduction and Cut-Off Estimation

Of the 143 patients, 127 could be used to calculate the relation between palbociclib exposure and a dose reduction (10 patients without complete intake and follow-up history and 6 patients who began at dose lower than 125 mg/d). Of these 127 patients, 75 were not subject to a dose decrease during treatment.

**Figure 5:**
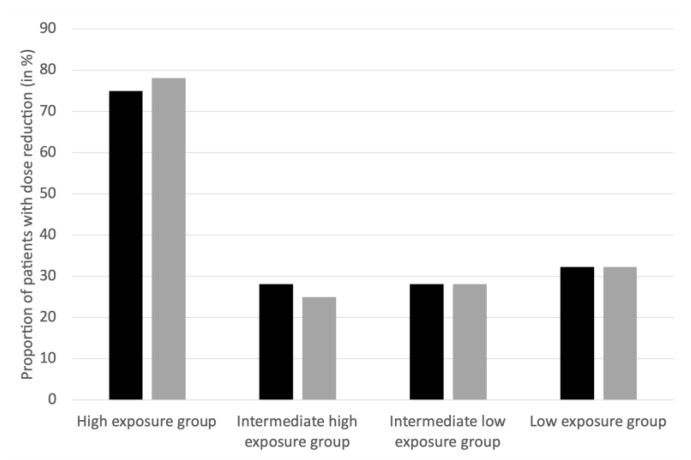
Proportion of patients with dose reduction. Black bars represent AUC (Area under the curve (in mg.h/L)) and gray bars represent C_resSS_ (Residual concentration at the steady state (in µg/L)). High exposure group (*n* = 32): 2.77 < AUC < 5.63 or 95 < C_resSS_ < 204, intermediate high exposure group (*n* = 32): 2.17 < AUC < 2.76 or 70 < C_resSS_ < 94, intermediate low exposure group (*n* = 32): 1.76 < AUC < 2.16 or 53 < C_resSS_ < 69, and low exposure group (*n* = 31): 1.12 <AUC < 1.75 or 27 < C_resSS_ < 52.

For both the estimated AUC and estimated C_resSS_, we observed that the proportion of patients with a dose reduction was more important in the highest exposure group (estimated AUC > 2.77 mg.h/L or estimated C_resSS_ > 95 µg/L): 75% to 78.1% of the most exposed patients had a dose decrease, compared to 25% to 32.3% in the other groups (Figure 5).

## 4. Discussion

Neutropenia is the most common adverse event during treatment with palbociclib [1,2,3,5,6,15]. In the event of grade 3 (ANC < 1 G/L) and grade 4 (ANC < 0.5 G/L) neutropenia, the treatment is interrupted, and the dose may be reduced [3,5]. Investigation of ANC kinetic during palbociclib treatment may provide a better understanding of the exposure-toxicity relationship and allow us to predict which patients are more at risk. This study is the first PK/PD model of ANC time-course in real-life patients treated by palbociclib during one year.

To our knowledge, the only popPK model of palbociclib built from rich data is the one developed by Sun [16]. This model was built with data from phase I and I/II clinical trials (183 patients with an average of 10.6 observations per patient). This model is a bicompartmental model with an absorption lag time and a first-order constant of absorption. With our data, the estimation of either lag time or bicompartmental model was impossible. Indeed, only 4% of the samples were taken before the 2nd hour after the last dose and 5.5% after the 30th hour after the last dose. This lack of early and late observations makes it impossible to estimate parameters associated with the second compartment. Moreover, in contrast to the model proposed by Royer [14], we were not able to estimate the value of the volume of distribution and the variability of the absorption constant. Nevertheless, we observeda good fit of the model to the data as shown in Figure 2B.

During the covariate step, in addition to ALP, age, weight, creatininemia and Cl_cr_ (according to the Cockcroft–Gault formula) were significantly linked to Cl/F IIV when added solely. However, during the backward step, only the Cl_cr_ (and ALP) showed a significant impact. This may be explained by the fact that Cl_cr_ considers all the other covariates. Although renal elimination is not the major elimination route of palbociclib, study showed a relationship between renal insufficiency (according to the Cl_cr_ calculated by the Cockcroft–Gault formula) and palbociclib AUC, which quantifies the exposure to palbociclib. A decrease in Cl_cr_ leads to an increase in the AUC as a consequence of a decrease in the elimination of palbociclib [17]. According to its main route of elimination (i.e., hepatic [3,5]), the model also showed a relationship between ALP and palbociclib elimination. The higher the ALP, the lower the elimination. An increase in ALP can be explained by liver damage secondary to breast cancer or during liver disease [18].

Parameters estimated to describe the ANC time course were Base (2.92 G/L; 29.6% IIV), Slope (0.0011 L/µg; 28.8% IIV), Mean Transit Time (MTT; 5.29 days; 17.9% IIV) and γ (0.102). They are close to most of the previous PK/PD models for other compounds, but also to the model by Sun [9,19,20]. The fact that a patient experiences metastases and/or has already had chemotherapy and/or long-term hormone therapy and/or radiotherapy could suggest that the patient suffers from an advanced disease and therefore a poorer general condition that could lead to a higher risk of neutropenia [21]. After modelling the ANC kinetic, these covariates did not show a significant impact in our PK/PD model, nor the type of concomitant hormone therapy. The fact that these covariates did not emerge as significant indicates that these characteristics are not of major interest in the choice of palbociclib dosage. This observation is in agreement with several other pre-existing models that did not show relationships between these covariates and ANC kinetics during cytotoxic dosing [9,19,20].

The only covariate that emerged as significant in the PK/PD model was the effect of age on the patient circulating neutrophils at baseline. The older the patient, the more circulating neutrophils at the beginning of treatment. In several existing models, a negative correlation between ANC at initiation and albuminemia was highlighted: the lower the albuminemia, the higher the ANC. According to Schmitt et al. [12], the relationship between albuminemia and circulating neutrophils can be explained by the fact that low albuminemia is a sign of inflammation and therefore of increased neutrophils synthesis. In the paper by Sun, the impact of albuminemia on ANC was also evidenced, with a parameter associated with baseline albuminemia of −1.03. In our data, albuminemia did not show a significant impact on ANC at initiation, whereas age did. In our population, there is a negative correlation between age and albuminemia (data not shown), which may explain why age has an impact on the parameter Base. Indeed albuminemia showed a significant impact in the forward step but not in the backward step. Part of the effect of albuminemia must be accounted for by age. Sun also showed the impact of gender on the Base value. Our study cannot evidence this correlation because all our patients were female. Nevertheless, Base value is lower than the one for women estimated by Sun (2.92 versus 3.63 G/L). Our study is based on real life patients and not on patients selected for clinical trials. Therefore, we may treat patients in poorer general condition and with lower ANC. Among other parameters independent of treatment intake, MTT and γ do not differ too much from pre-existing models [8,9,19,20].

Regarding the limitations of this work, we assume a good compliance of the patient, and an error-free completion of patients’ information. However, as we conducted a retrospective study, some patient records did not include all the blood samples recommended by the French drug agency (ANSM). The poor estimation of the extreme values of our data can be explained by the low number of observations for patients with grade 3 (25.7%) and grade 4 (1.6%) neutropenia. The small number of patients may also be a limitation in the determination of covariates. A last limitation of our work is the absence of PK/efficacy analysis. A non-significant trend between mean trough concentrations and progression-free survival was highlighted [22]. Yet, our data were not mature enough to draw any firm conclusion for efficacy.

Because no formal target concentration exists for palbociclib, Mueller-Schoell et al. suggest using the mean trough concentration observed in the clinical trials (61 µg/L) as a target [23]. This concentration leads to a risk of grade 3 neutropenia of approximately 31% and of grade 4 neutropenia of approximately 2%. As patients experiencing severe palbociclib-induced neutropenia are at low risk of infection [24], we have refined this therapeutic target to a higher value. Based on our simulations, in order to avoid a risk of grade 4 neutropenia greater than 20%, estimated C_resSS_ should not exceed 100 µg/L. This is confirmed, in real life, by the fact that patients with C_resSS_ > 95 µg/L benefit of nearly 3 times more dose reductions than patients with lower exposure (Figure 5). Thus, a C_resSS_ lower than 100 µg/L seems reasonable to avoid severe toxicity. It should be noted that, as not all measured concentrations were trough at steady-state, C_resSS_ was estimated for all patients. Thus, all our interpretations are based on C_resSS_ estimated from our popPK model.

## 5. Conclusions

A popPK model and a semi-mechanistic PK/PD model constructed from general population data were presented. Interindividual variability in Cl/F was partially explained by Cl_cr_ and ALP concentration. Interindividual variability in ANC at baseline was also partially explained by age (without any physiological explanation). Based on our simulations, an estimated palbociclib C_resSS_ below 100 µg/L would limit the risk of grade 4 neutropenia below 20% and should not be exceeded. In order to obtain a full target (i.e., a low boundary in addition to this high boundary), exposure/efficacy relationships should be investigated.

## Figures and Tables

**Table 1:** Patients’ demographic and biological characteristics. ALP: alkaline phosphatase; ALAT: alanine aminotransferase; ASAT: aspartate aminotransferase; Cl_cr_: creatinine clearance; γ-GT: γ-glutamyltranspeptidase; popPK: population pharmacokinetic; PK/PD: pharmacokinetic/pharmacodynamics.

	Population Used to Build the popPK Model (*n* = 143)		Population Used to Build the PK/PD Model (*n* = 128)	
	Median (min–max)	Number	Median (min–max)	Number
Age (years)	69 (40–92)		63 (40–92)	
Serum creatinine (µmol/L)	68.0 (31.0–301.8)		68.0 (31–169.7)	
Weight (kg)	67 (37–140)		66 (37–140)	
Clcr (according to Cockcroft–Gault formula) (mL/min)	71.6 (22.1–282.3)		73.5 (22.1–282.3)	
ALAT (UI/L)	18 (6–237)		17 (6–237)	
ASAT (UI/L)	23 (12–205)		22 (12–205)	
γ-GT (UI/L)	29 (9–1113)		28 (9–1113)	
ALP (UI/L)	89 (11–819)		81 (11–644)	
Lactate dehydrogenase (UI/L)	224 (85–675)		216 (85–675)	
Total bilirubin (mg/L)	5.0 (1.5–27.2)		4.7 (1.5–27.2)	
Albumin (g/L)	39.9 (20.0–48.0)		39.0 (20.0–48.0)	
Plasma protein (g/L)	70.6 (53.0–98.0)		69.0 (53.0–88.0)	

**Table 2:** Patients’ treatment and disease characteristics. NK: not known; popPK: population pharmacokinetic; PK/PD: pharmacokinetic/pharmacodynamic.

	Population Used to Build the popPK Model (*n* = 143)		Population Used to Build the PK/PD Model (*n* = 128)	
	Median (min-max)	Number	Median (min-max)	Number
Number of previous chemotherapies	2 (0–12)		2 (0–12)	
Long-term hormonotherapy				
Yes		93 (65.0%)		90 (70.3%)
No		40 (28.0%)		38 (29.7%)
NK		10 (7.0%)		0 (0.0%)
Previous radiotherapy				
Yes		73 (51.0%)		73 (57.0%)
No		60 (42.0%)		55 (43.0%)
NK		10 (7.0%)		0.0 (0.0%)
Metastases				
Yes		108 (75.5%)		105 (82.0%)
No		25 (17.5%)		23 (18.0%)
NK		10 (7.0%)		0.0 (0.0%)
Concurrent hormonotherapy				
Fulvestrant		48 (33.6%)		45 (35.2%)
Letrozole		71 (49.6%)		69 (53.9%)
Others		14 (9.8%)		14 (10.9%)
NK		10 (7.0%)		0 (0.0%)

**Table 3:** Estimated parameters of the final popPK model. ALP: alkaline phosphatase; Cl/F: palbociclib apparent oral clearance; CV: coefficient of variation; Cl_cr_: creatinine clearance according to Cockcroft–Gault formula; IIV: inter individuality variability; k_a_: Palbociclib absorption rate; med: median; RSE: relative standard error; T_lag_: Palbociclib absorption lag time; V/F: palbociclib apparent volume of distribution.

	Population PK Model without Covariates		Final Population PK Model with Covariates		Bootstrap of the Final Population PK Model ^a^		Consequences of Covariates
Objective Function Value	1775.75		1734.37				
	Parameter	RSE (%)	Parameter	RSE (%)	Parameter	2.5th–97.5th percentiles	
Cl/F (L/h)	57.42	3.2	57.13	2.8	57.21	[54.19–60.42]	
Cl_cr_ (med = 71.6 mL/mn) on Cl/F	.	.	0.44	15.1	0.46	[0.35–0.57]	Cl/F increase when Cl_cr_ increase
ALP (med = 88.6 UI/L) on Cl/F	.	.	−0.14	34.1	−0.15	[−0.24–−0.05]	Cl/F increase when ALP decrease
V/F (L)	1580	.	1580	.	.	.	
k_a_ (h^−1^)	0.187	21.2	0.187	22.2	0.18	[0.17–0.19]	
T_lag_(h) (fix)	0.658	.	0.658	.	.	.	
Cl/F IIV (CV %)	41.4	6.5	32.6	9.3	30.7	[21.8–37.1]	
Additive error (µg/L)	10.12	19.6	13.84	20.5	14.70	[6.46–22.48]	

^a^ Estimates are presented with the median and 2.5th–97.5th percentiles values of the bootstrap (*n* = 1000).

**Table 4:** Estimated parameters of the final PK/PD model. CV: coefficient of variation; IIV: inter individuality variability; med: median; MTT: mean transit time; RSE: relative standard error.

	Population PK/PD Model without Covariates		Final PK/PD Model with Covariates		Consequences of Covariates	Bootstrap Simulation	
Objective Function Value	2410.37		2400.90				
	Parameter	RSE (%)	Parameter	RSE (%)		Median Parameter ^a^	2.5th–97.5th Percentiles
Base (G/L)	2.94	3.70	2.92	3.55		2.95	[2.75–3.16]
Slope (L/µg)	0.0011	6.60	0.0011	6.46		0.0011	[0.0009–0.0014]
MTT (days)	5.32	4.81	5.29	4.93		5.36	[4.61–6.30]
Gamma	0.109	7.52	0.103	7.01		0.104	[0.085–0.137]
Base IIV (CV %)	31.8	8.67	29.6	9.21		29.0	[24.6–33.1]
Age on Base (med = 63.7 years)			0.465	34.00	Base increases with age increase	0.51	[0.23–0.78]
Slope IIV (CV %)	26.4	12.80	28.8	11.70		28.0	[21.2–34.1]
MTT IIV (CV %)	18.1	21.00	17.9	21.10		19.0	[10.0–33.0]
Exponential error	0.34	2.40	0.34	2.02		0.33	[0.31–0.36]

^a^ Estimates are presented with the median values of the bootstrap (*n* = 100).

## Data Availability

Data available on request.

## Supplementary Materials

The following are available online at https://www.mdpi.com/1999-4923/13/10/1708/s1. Figure S1. Numerical predictive checks (NPC) of the palbociclib final population pharmacokinetic model. Figure S2. Cumula tive distribution functions of the normalised prediction distribution errors (NPDE) of the final Palbociclib population pharmacokinetic model. Figure S3. Probability density function of the normalised prediction distribution errors (NPDE) of the final Palbociclib population pharmacokinetic model. Figure S4. Prediction corrected visual predictive checks (VPCc) of the final Palbociclib pharmacokinetic/pharmacodynamic model. Figure S5. Cumulati ve distribution functions of the normalised prediction distribution errors (NPDE) of the final Palbociclib pharmacokinetic/pharmacodynamic model. Figure S6. Probability density function of the normalised prediction distribution errors (NPDE) of the final Palbociclib pharmacokinetic/pharmacodynamic model.
