# Supplementary material for: Pharmacokinetic/Pharmacodynamic Model of Neutropenia in Real-Life Palbociclib-Treated Patients
Source: Pharmaceutics. 2021 Oct 16;13(10):1708. doi: 10.3390/pharmaceutics13101708 (PMC8537267; doi:10.3390/pharmaceutics13101708)
Supplement: Supplementary file 1 [file pharmaceutics-13-01708-s001.zip › pharmaceutics-1393351 - corrected final.pdf]

pharmaceutics

MDPI

Supplementary Materials: Pharmacokinetic/Pharmacodynamic Model of Neutropenia in Real-Life Palbociclib-Treated Patients

Alexandre Le Marouille, Emma Petit, Courèche Kaderbhaï, Isabelle Desmoulins, Audrey Hennequin, Didier Mayeur, Jean-David Fumet, Sylvain Ladoire, Zoé Tharin, Siavoshe Ayati, Silvia Ilie, Bernard Royer and Antonin Schmitt

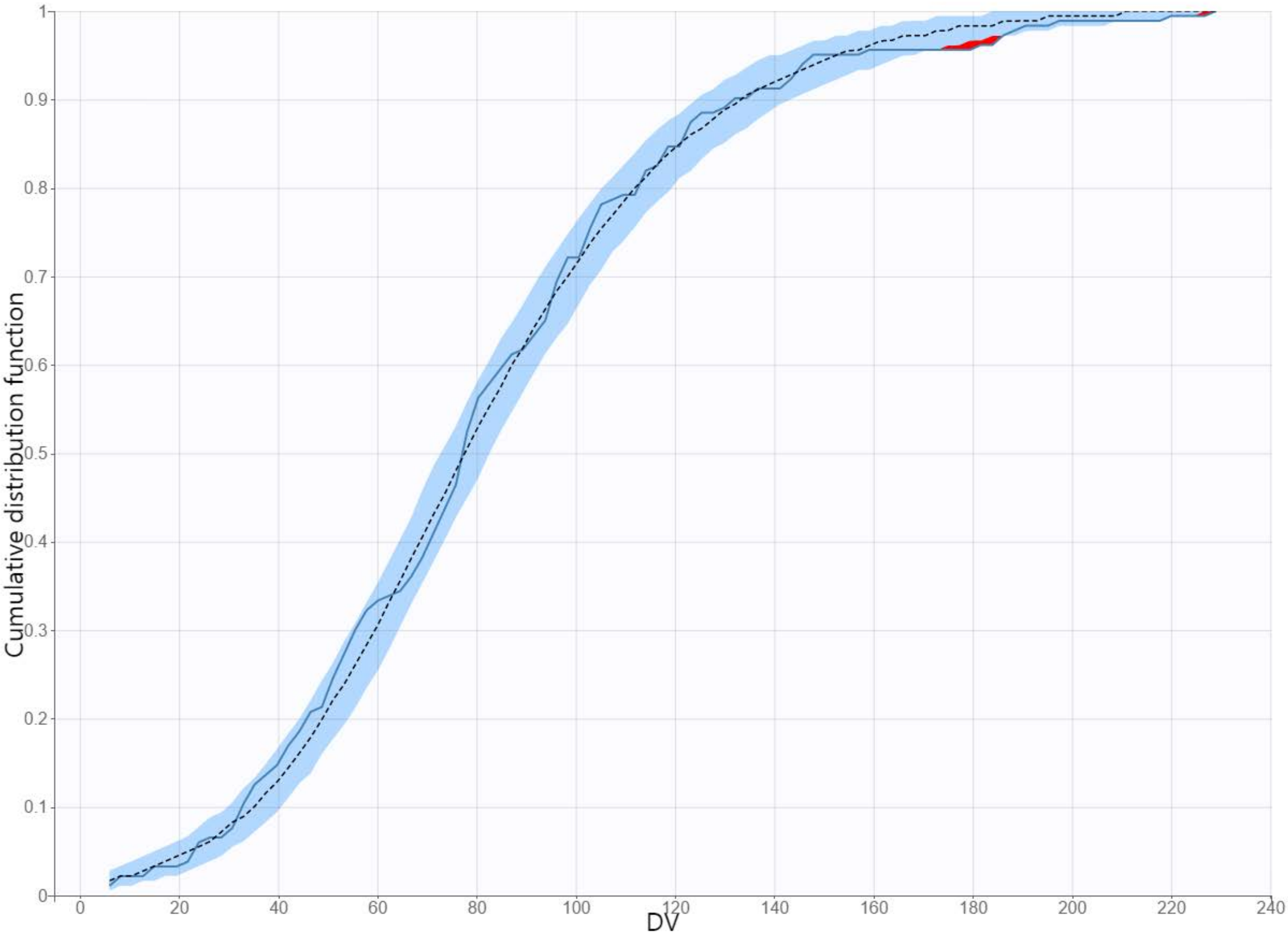

Figure S1. Numerical predictive checks (NPC) of the palbociclib final population pharmacokinetic model. DV: palbociclib concentrations in µg/L. The solid blue line represents the cumulative distribution function of the observed data. The dash black line represents the cumulative distribution function of the simulated data with the population pharmacokinetic model. The blue area represents the 90% confidence intervals of the cumulative distribution function of the simulated data. The red areas represent the outliers (empirical distribution outside of the prediction intervals).

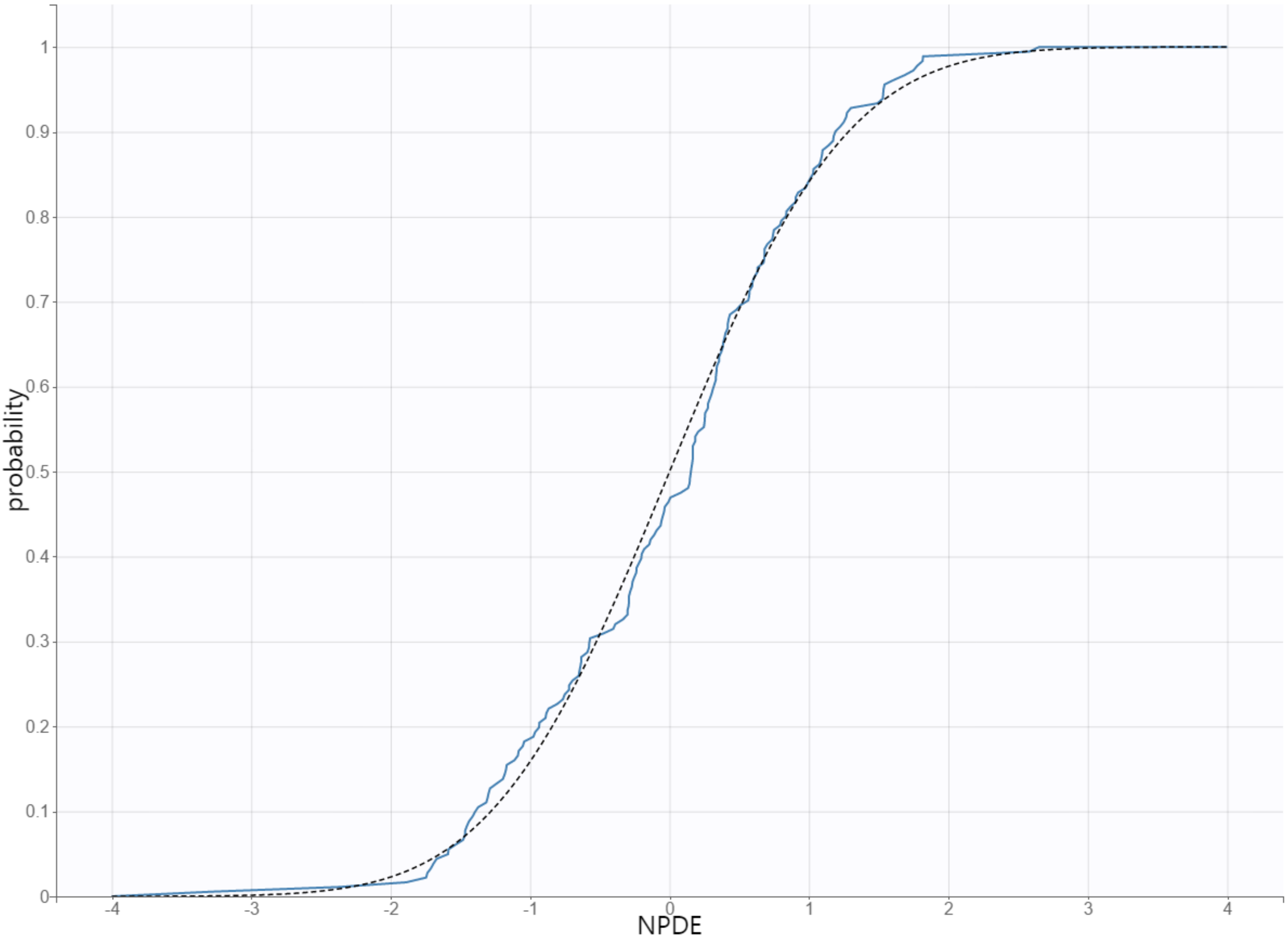

Figure S2. Cumulative distribution functions of the normalised prediction distribution errors (NPDE) of the final Palbociclib population pharmacokinetic model. The dash line represents the theoretical cumulative distribution of the NDPE defined by the population pharmacokinetic model and the blue line represents the cumulative distribution of the empirical NDPE.

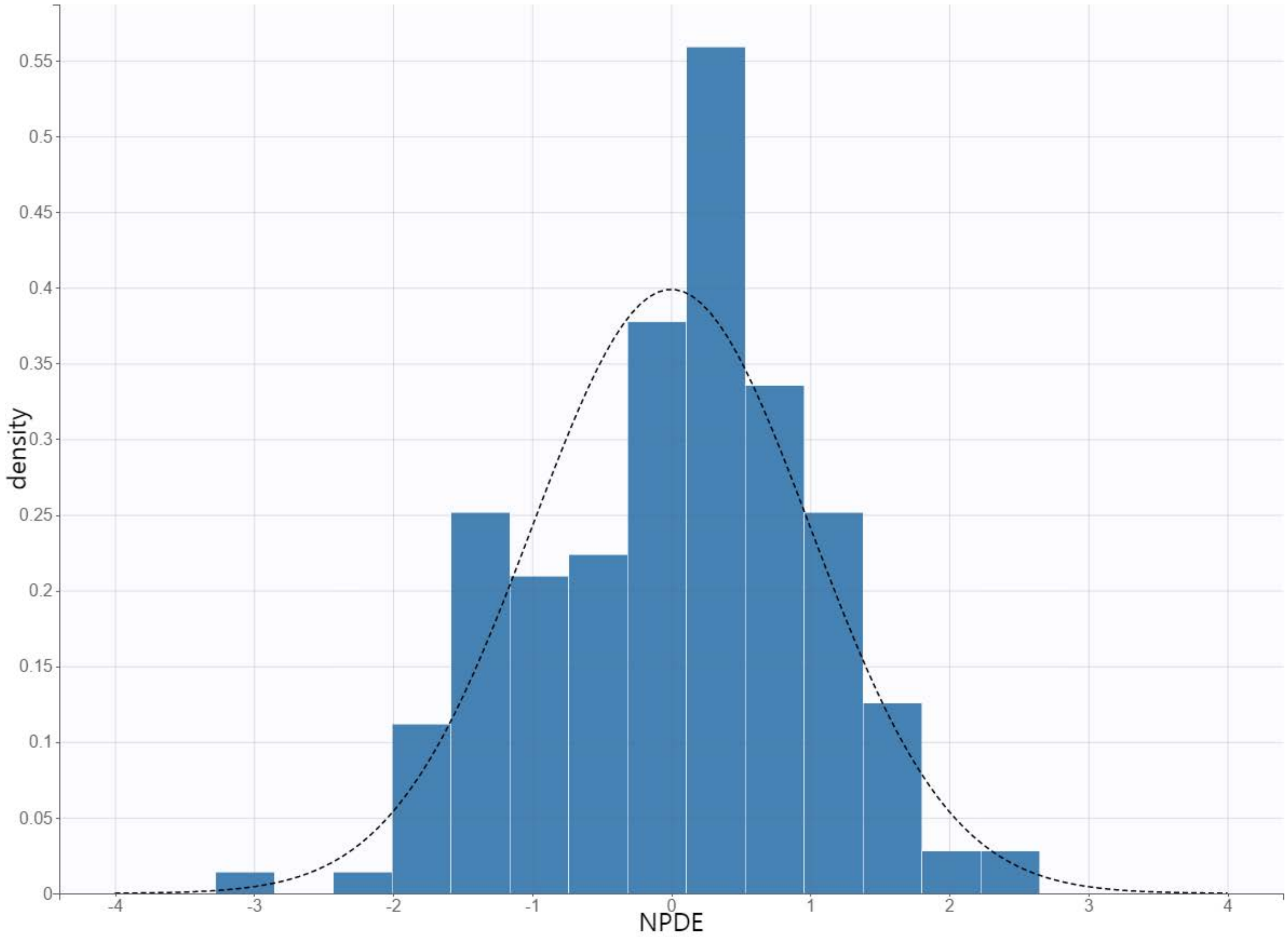

Figure S3. Probability density function of the normalised prediction distribution errors (NPDE) of the final Palbociclib population pharmacokinetic model. The dash line represents the theoretical distribution of the NDPE defined by the population pharmacokinetic model and the blue bars the empirical distribution of the NPDE.

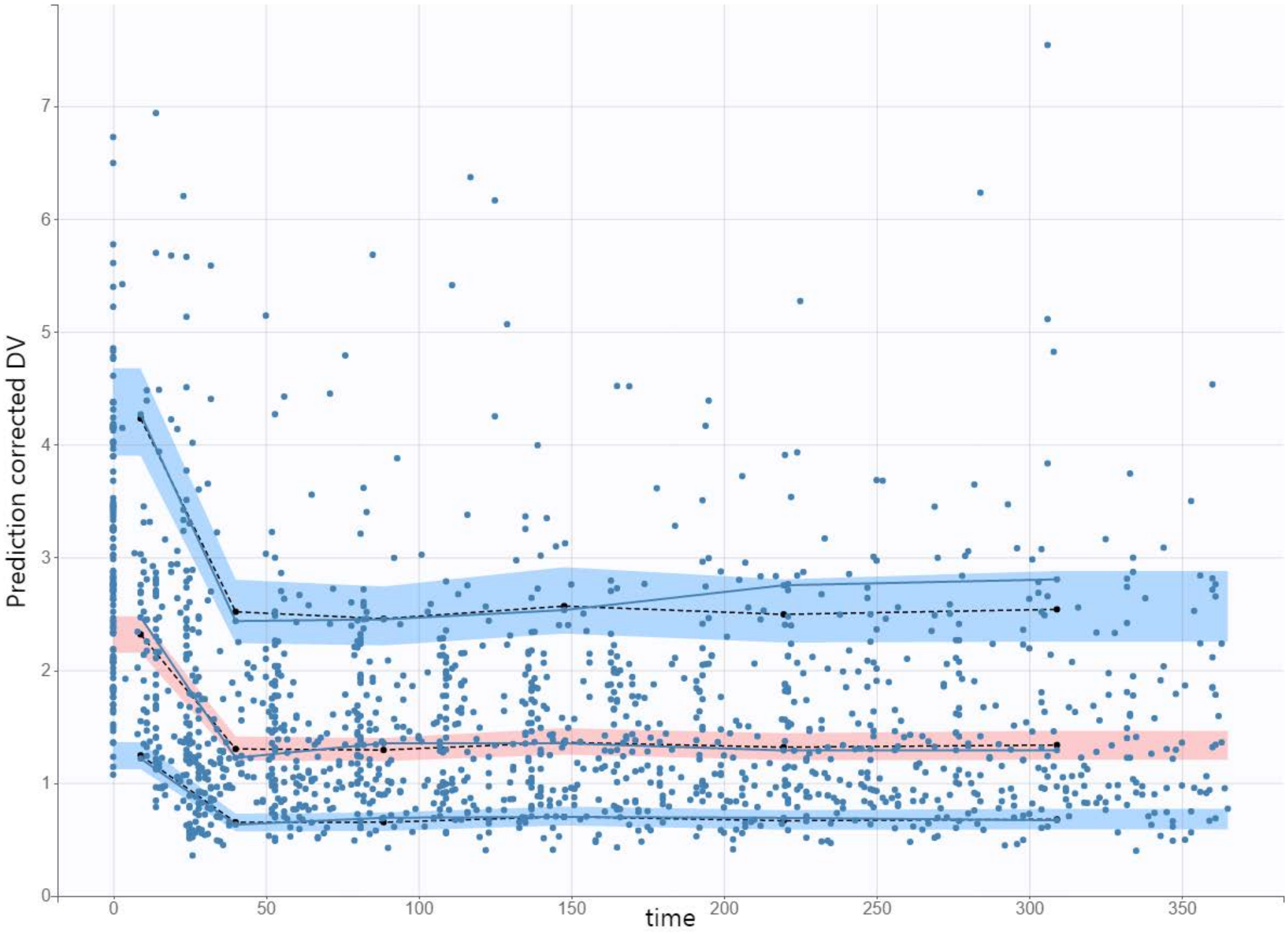

Figure S4. Prediction corrected visual predictive checks (VPCc) of the final Palbociclib pharmacokinetic/pharmacodynamic model. Prediction corrected DV: Absolute Neutrophil Counts (× 10^9/L). The solid blue line represents the 10th, 50th, and 90th percentiles of the observed data. The blue areas represent the 90% confidence intervals (CI90) around the 10th and 90th percentiles and the pink area represent the CI90 of the 50th percentiles of the simulated data. The observed plasma concentrations are represented by blue dots.

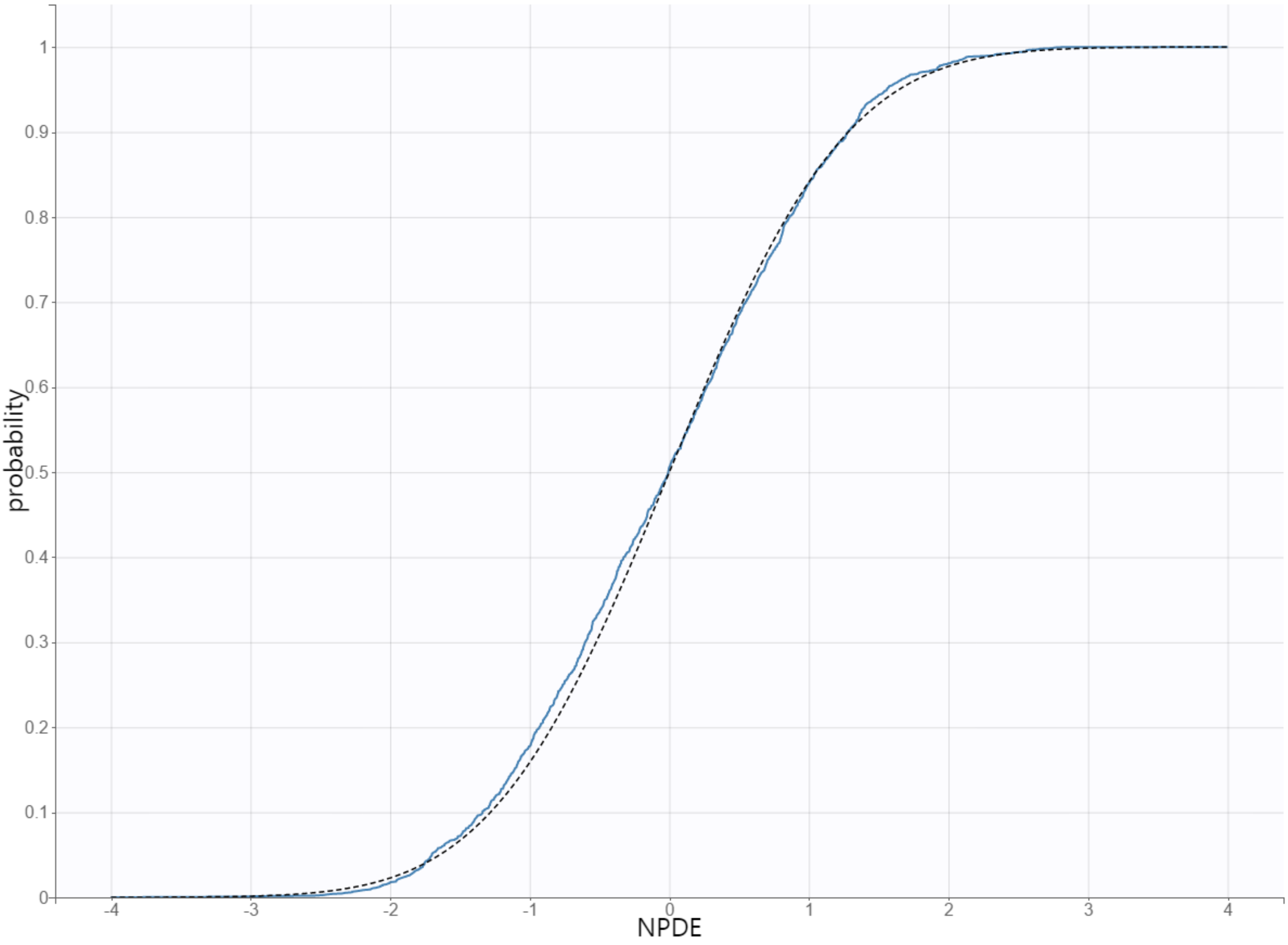

Figure S5. Cumulative distribution functions of the normalised prediction distribution errors (NPDE) of the final Palbociclib pharmacokinetic/pharmacodynamic model. The dash line represents the theorical cumulative distribution of the normal distribution defined by the final pharmacokinetic/pharmacodynamic model and the blue line represents cumulative distribution of the empirical NDPE.

Supplementary

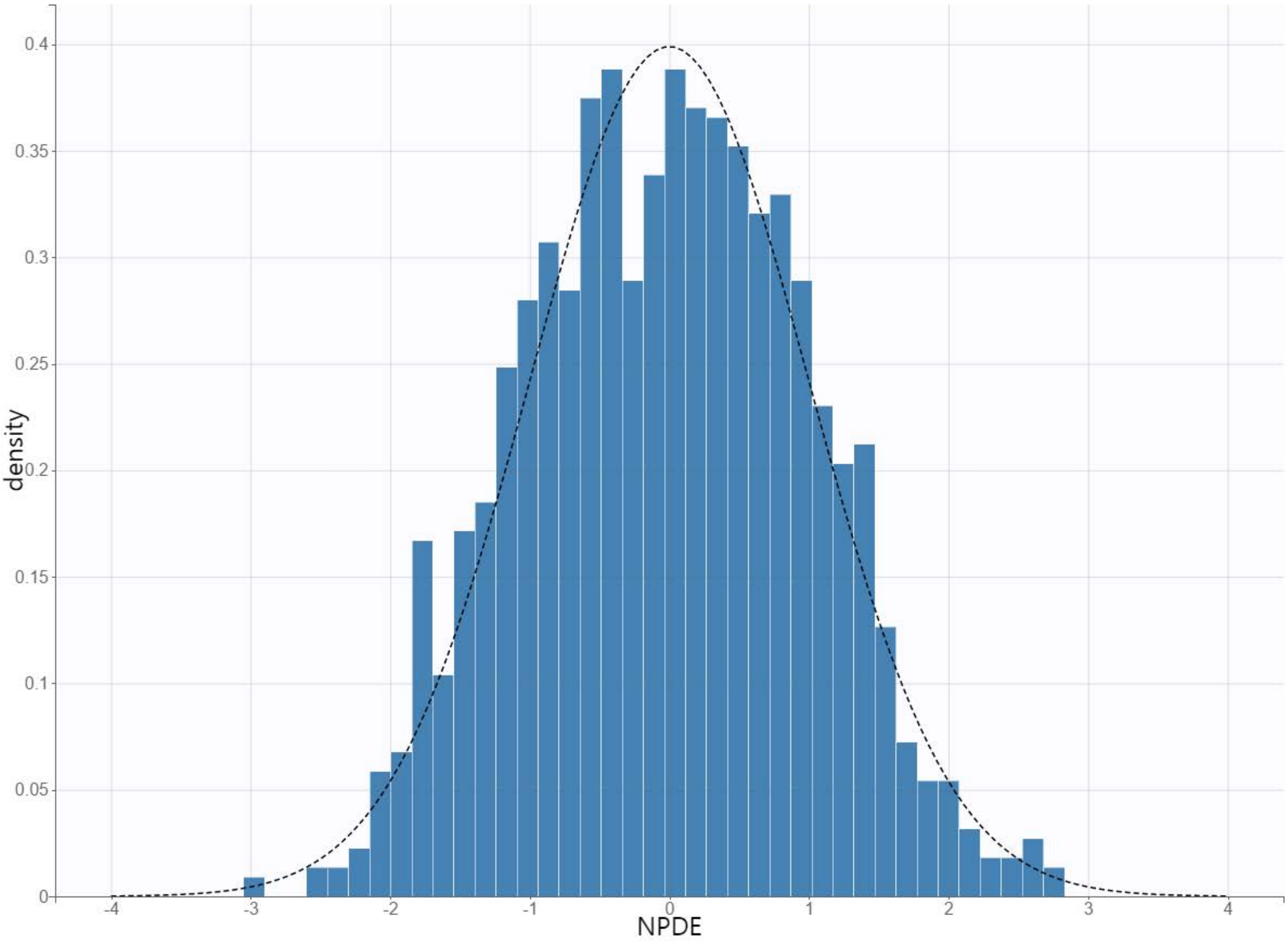

Figure S6. Probability density function of the normalised prediction distribution errors (NPDE) of the final Palbociclib pharmacokinetic/pharmacodynamic model. The dash line represents the theoretical distribution of the NDPE defined by the pharmacokinetic/pharmacodynamic model and the bars the empirical distribution of the NPDE.
